# Text mining for biology - the way forward: opinions from leading scientists

**DOI:** 10.1186/gb-2008-9-s2-s7

**Published:** 2008-09-01

**Authors:** Russ B Altman, Casey M Bergman, Judith Blake, Christian Blaschke, Aaron Cohen, Frank Gannon, Les Grivell, Udo Hahn, William Hersh, Lynette Hirschman, Lars Juhl Jensen, Martin Krallinger, Barend Mons, Seán I O'Donoghue, Manuel C Peitsch, Dietrich Rebholz-Schuhmann, Hagit Shatkay, Alfonso Valencia

**Affiliations:** 1 Stanford University, 318 Campus Drive, Stanford, California, 94305-5444, USA; 2 University of Manchester, Michael Smith Building, Oxford Road, Manchester, M13 9PT UK; 3 Jackson Laboratory, 600 Main Street, Bar Harbor, Maine, 04609, USA; 4 Bioalma, Ronda de Poniente 4, Bajo C, 28760 Tres Cantos, Madrid, Spain; 5 Oregon Health & Science University, 3181 SW Sam Jackson Park Road, Portland, Oregon, 97239 USA; 6 Science Foundation Ireland, Dublin, Ireland; 7 EMBO, Postfach 1022.40, Heidelberg, D-69117 Germany; 8 Jena University, Fuerstengraben 30, Jena, D-07743, Germany; 9 MITRE, 202 Burlington Road, Bedford, Massachusetts, 01730 USA; 10 European Molecular Biology Laboratory, Meyerhofstrasse 1, Heidelberg, D-69117 Germany; 11 NNF Center for Protein Research, Panum Institute, Copenhagen, Denmark; 12 CNIO, C/Melchor Fernandez Almagro, 3, Madrid, E-28029 Spain; 13 Erasmus Medical Center and Leiden University Medical Center, Leiden, Bldg. 2, Einthovenweg 20, Leiden, 2300 RC, The Netherlands; 14 Swiss Institute of Bioinformatics, Quartier Sorge-Batiment Genopode,, Lausanne, 1015 Switzerland; 15 EBI, 1, Wellcome Trust Genome Campus, Hinxton, CB10 1SD, UK; 16 School of Computing, Goodwin Hall, Queen's University, Kingston, Ontario, K7L 3N6, Canada

## Abstract

This article collects opinions from leading scientists about how text mining can provide better access to the biological literature, how the scientific community can help with this process, what the next steps are, and what role future BioCreative evaluations can play. The responses identify several broad themes, including the possibility of fusing literature and biological databases through text mining; the need for user interfaces tailored to different classes of users and supporting community-based annotation; the importance of scaling text mining technology and inserting it into larger workflows; and suggestions for additional challenge evaluations, new applications, and additional resources needed to make progress.

## Introduction

This supplement has focused on progress in text mining applied to biology, and genomics in particular, as reflected in the BioCreative II results. There is a growing demand to 'translate' information from text into more computable forms and to cross-link the information with relevant biological databases. This linkage has the potential to improve the connection between the annotations in biological databases and the supporting evidence contained in the literature. Current biological databases rely heavily on expert human curation, which requires that PhD level biologists read the (relevant) literature carefully, extract specific kinds of information, and encode each snippet of information into an entry in a database using an ontology or controlled vocabulary (see article by Chatr-aryamontri and coworkers [1] in this supplement). Given the growing volume of literature and new high-throughput methods, it is becoming urgent to provide tools that can reduce time and cost of curation, increase consistency of annotation, and provide the linkages to supporting evidence in the literature that make the annotations useful to researchers. Indeed, the distinction between biological databases and the literature is becoming increasingly blurred [2-4], and there is active discussion about whether capture of information from free text can be done before publication or extracted from the literature after publication. In addition, we are seeing the emergence of new tools to aid in massive extraction of information from both literature and biological databases (for example, WikiProfessional [5]) or on-demand extraction of information from the literature (Information Hyperlinked Over Proteins [iHOP] [6]). These tools will provide improved access to different classes of users, who need different types and granularities of information, ranging from retrieval of relevant articles, to identification of passages or individual sentences, to phrases or biological facts, generally encoded in a controlled vocabulary or ontology.

We have invited a group of leading scientists from biology, pharmacology, bioinformatics, and computer science to provide their views on the importance of text mining for biology, the utility of current systems, and where to go next. To frame the discussion, we provided the contributors with a short set of questions related to how text mining can improve information access. As the organizers of BioCreative, we are specifically interested in how community challenges can direct the research community toward biologically relevant problems. While we believe that formal evaluation on carefully prepared training and test collections is useful to drive research, we also believe that it is critical to move the tools towards the end users in the bioinformatics and biology communities. We value the contributions provided by the contributors in response to the questions below.

### The questions

• In light of the BioCreative results, do you think that text mining can help to make biological knowledge more accessible? If so, how? If not, then why not?

• What are the next steps that the bioinformatics community needs to take to provide better access to biological knowledge?

• In your view, how can the BioCreative challenge evaluations contribute to solving these problems? What problems should the next BioCreative tackle?

### The major themes

Not surprisingly, there are a number of common themes that run through the responses. The respondents emphasized the need to identify the different classes of end users and to apply the technology to the pressing problems facing these users to manage access to the rapidly expanding literature. The themes fall into several broad categories.

#### Fusing literature and biological databases through text mining

Text mining has the potential to make accessible the rich information contained in free text, and combine evidence mined from text with other sources of evidence from biological databases. However, realization of this potential will require active engagement with publishers and content providers, to make full text articles available, to link content into existing resources, and to make the meta-data with these linkages readily accessible.

#### Interactivity and user interfaces

The end users (for instance, researchers from bioinformatics, biology, biomedicine, and pharmacology) must find the interface intuitive and usable with little or no understanding of text mining and the natural language processing technologies that lie behind it. Another class of users (the 'content providers', including curators) will require an interactive interface that lets them change annotations, drill down for evidence, link across resources, and create new information resources to capture new concepts as they are discovered. There is increasing interest in community-based annotation, and in providing the tools to support this.

#### Tool integration

Several contributors make the point that text mining tools will be most effective if they are integrated into a larger workflow. The tools must scale to handle real collections (for example, all of Medline). Integration will also require stable standards for exchange and integration of information derived from text mining.

#### Text mining resources

There is still the need for additional resources (lexicons, terminology standards, ontologies, and additional challenge evaluations). Several contributors make detailed recommendations for future challenge evaluations.

#### Recommendations for future BioCreatives

Many of the opinions contain suggestions for future directions. These include broadening the range of entities beyond genes and proteins to include, for example, chemicals or diseases; broadening the types of relations, including complex relations, such as genotype-phenotype relations; capturing biological evidence better and differentiating well known from novel findings; and broadening the types of information mined to include full text, images, supplementary materials, and even Wikis and blogs. There are also a number of new applications suggested, including automated generation of datasets to support curation resources and ontology building; automatically generated abstracts and summaries; and collaborative 'curation' or editing (folksonomies) that can combine both mined information and expert-generated relations. An important role for challenge evaluations is to provide opportunities for closer collaboration between developers of text mining tools and end users.

### Selecting new challenge tasks

The BioCreative organizers will continue to work closely with the diverse communities involved. The goal is to select tasks based on available datasets that meet the following criteria. These datasets would:

• Address the requirements of real users.

• Contribute to real data needs, ideally where a (partial) 'gold standard' is already available, for example from an expert-curated resource.

• Approximate real uses as closely as is consistent with an ability to evaluate and compare results.

• Use commonly accepted standards for representation.

• Be made available - including results, data, and tools - to the larger community for both further research and application.

### The structure of this report

There are 12 responses in the remainder of the article. The first two (Gannon/Grivell and Peitsch) provide a broad context and address the relationship between text mining and biomedical literature; the second two (Mons and O'Donoghue/Jensen) provide the perspective of developers of large scale resources that use text mining; the third set comes from curators and 'consumers' of biological information (Altman, Blake, and Bergman); the final set of opinions comes from text mining researchers in both industry and academia (Blaschke, Shatkay, Cohen/Hersh, Rebholz-Schuhmann, and Hahn). These latter contributions in particular make some very specific suggestions about possible directions for future BioCreatives. In the Conclusion section (below), the organizers (Hirschman, Krallinger, and Valencia) highlight the key challenges for text mining, particularly regarding the 'triangle' of publications, databases, and users/developers.

## The contributors and their opinions

### Frank Gannon and Les Grivell: future research paradigms and the demands on scientific publications

*Frank Gannon is currently Director General of Science Foundation Ireland (SFI). He is also Senior Editor of EMBO Reports and previously was the Executive Director of EMBO. He is a molecular biologist and was a senior Scientist at EMBL, where his research group worked on the control of expression by the estrogen receptor*.

*Dr Les Grivell is Manager for the EMBO publications at the EMBO office in Heidelberg, Germany. His background is in molecular biology and his current interests are in computational and systems biology, text mining, and scientific publishing*.

In the not so long distant past, research publications usually arose from work in a single laboratory and were presented in a printed journal. At that time it was not unusual for both authors and readers to know all of the relevant related studies, such that any newly published paper represented an update of linearly derived information.

Things changed radically from the onset of a data intense 'omics' era. Since then it has become close to impossible to 'know' everything. Initially, it might have seemed unnecessary to do so, because research output was focused close to a particular target topic (for instance, gene) and the main aim was to avoid the embarrassment of resequencing a gene that had already been included in another study. For that step it was necessary to have access to and add to relevant databases. New users entered this zone with some concern, but soon learned that the information there could significantly accelerate the isolation of a gene of interest in the laboratory. Access to the sequence was more or less all that was needed and saved a lot of time and work. Use of the databases grew.

As the tsunami of data increased and the diversity of the types of information exploded, a series of significant changes inevitably followed. For scientists it became essential, rather than simply clever, to integrate fully the data of others into their studies. It also challenged the journals to change from a centuries old mode of dissemination to one that - at least in the beginning - meant providing information in both print and electronic forms. As time rolls on, it is becoming increasingly obvious that the process of publication as we used to know is becoming an anachronism. Paper and print constitute constraints of space and structure. Those who are driving forward thinking in the area have developed an understanding of the need for a new and further evolved publication paradigm. It is easy to anticipate that publications will be effectively an extended, computer-readable abstract with linkages to different new results and relevant archived data. Also, in conjunction with this development, the growth of Wiki and other Web2.0 technologies will lead to a rapid evolution of new ways of communicating and exchanging information relating to research data and their interpretation.

In this next phase of publication there will be a greater appreciation of the importance of the archival role of publications. This will arise as postgenomic work and screening of large bodies of data lead research into unexpected areas and topics that are far from the original start point of research in a given laboratory. Today it is obvious to most that the focus of attention should be on the underlying biological problem rather than on what happens to the protein that has been worked on perhaps since the group leader started his/her laboratory. This creates different dynamics and new expectations of what publications should provide. The information that comes from previous (published) research must be not only accessible, but it must also be available in a manner that can be readily interrogated. For the primary data (for example, DNA sequence) this is taken charge of by central facilities such as the European Bioinformatics Institute (EBI) in Hinxton, UK. Assuming that this and similar groups continue to provide the right level of service, the needs of the scientific community will be provided for. However, much more information lurks in the literature, and it is often invisible from a search of the titles and abstracts in PubMed. Universal knowledge of the potential factors that impinge on a biological process is not available from reading the literature in a standard way, even if that includes Google-type searches. Significantly more information is hidden in the discussions or introductions of standard papers. There, the expert writing the paper draws together different strands of information that point the reader to potentially unexpected roles for a protein that might not have been the primary topic of the paper. This suggests the need to structure both data and text in such a way that it can be mined for connections of previously unlinked information. At the trivial level this might be an indication that is clearly hinted at by the author. At a more prospective level, the coincidence of reference to an unrelated entity by diverse papers could consolidate a concept that was previously too diffuse to take as a lead for more intense study.

With the growth in the datasets, both those simply deposited and those annotated by experts, the day should come when the most projects will be driven by and limited by the intelligent question posed by the researcher. An intense analysis of correctly structured full text publications will move the question to a point where a profound mining of the available databases, and in particular integration of different databases, such as DNA, protein, microRNA, protein-protein interactions, and so on, should enrich the preparatory phase. The final step of performing wet laboratory experiments then may fall soon into the category of confirmation rather than discovery.

For this to happen, however, there has to be agreement of standards that allow the easy movement from one platform to another and methods to allow easy mining of full text. Most of all, some changes in attitude are needed such that results deposited in text or database archives are viewed as the most important knowledge resources that have been generated by researchers and not simply as information that becomes out of date within a year of publication.

### Manuel C Peitsch: text mining as enabler in accessing biomedical knowledge

*Professor Manuel Peitsch is Head of Systems Biology at Novartis Institutes of BioMedical Research in Basel, Switzerland. His background is in biochemistry and computational life sciences and his current research focuses on text analytics for drug discovery, bioinformatics, proteomics, and systems biology*.

Text mining is emerging as a major enabler in accessing biomedical knowledge. Indeed, not only biological, but also chemical [7] and medical knowledge access represent major challenges to the research community, academic and industrial alike. It is obvious that the exponential increase in published science is posing major challenges to the community. Within this context, the recent BioCreative challenge addressed three central aspects of text mining that play a pivotal role in making biological knowledge more accessible and enabling applications, ranging from computer-assisted reading [8] to computational systems biology [9]. Based on this progress, we recommend that the community focus on the following specific technical challenges.

#### Creating computer-assisted reading applications

Establish applications that enable scientists to leverage the literature corpus more efficiently on demand. To this end, we should be witnessing further developments of concepts/tools such as iHOP [6,10], UltraLink [8], MEDIE[11], EBIMed [12], InFact [13], and so on.

#### Precomputing facts databases

Further develop precomputed facts databases, like the one behind iHOP and some commercial products. This might be done by using combinations of text mining techniques and machine learning to 'rewrite' parts of Medline abstracts in a formalized manner (for example, 'A does B when C'). Of course, over time, one could apply the same technology to full text. Eventually, this will lead to the formulation of the literature's core facts in a language that can be used for computer reasoning.

*Providing better ontologies and thesauri *to address issues such as higher precision, resolution of anaphoric references, disambiguation of terminology, acronyms, homographs and polysemy using context, and creation of intelligent Web crawlers/robots that leverage text mining.

*Building curation sets *to support ontologies, thesauri, and semantic networks. This is a crucial component to further improve text mining while keeping the curation costs within reasonable/manageable boundaries.

*Becoming the technology driver *to support the publishing revolution.

• By modernizing publishing practices, including structured abstracts, structured data tables, and/or database depositions.

• By modernizing publishing policies (mandatory use of DOI, establish SIN [Scientists Identification Number], and increased Open Access, especially for full text articles for text mining).

• By modernizing editing practices (definition of guidelines for gene names to support improved indexing; publishers/editors should ensure that these are done properly to ensure correct indexing)

• By negotiating with publishers; thus far, we have found this to be difficult. The breakthrough will probably come from uniting voices.

There are a number of steps that the bioinformatics community can take to provide better access to biomedical information, for example:

• Creating tools that hide the complexity and enable scientists to do the job themselves (hence the concept of computer assisted reading).

• Building multidisciplinary teams/task forces to achieve closer coupling of developers and users.

• Improving library sciences, to bring text mining closer to that branch; too many librarians still live in the pre-e world and even more so in the pre-text-mining era.

BioCreative could evolve to further define the challenges outlined above and become a more frequent event. This would certainly help accelerate progress and emphasize the importance of the field. Being more daring, one may imagine that BioCreative could become a foundation that could receive funds from private and public enterprises, which in turn could be given as prizes for certain grand challenges. For instance, I am thinking of the 'Board of Longitude' [14], which was formed in the 18th century to solve the problem of finding longitude at sea and to award a prize for specific achievements. Such a BioCreative Foundation could define some highly challenging goals and give a prize to the person or group who solves them.

### Barend Mons: from text to facts

*Dr Barend Mons is Associate Professor in Bio-Semantics at the Department of Medical Informatics, Erasmus Medical Centre, University of Rotterdam and (since 2005) at the Department of Human Genetics at the Leiden University Medical Centre, both in The Netherlands. His present activities mainly focus on international networking to realize a completely new form of computer-assisted distributed annotation and online knowledge discovery, in close collaboration between the University of Rotterdam, University of Leiden and Knewco, and largely based on the Knewco Knowlet*™ *technology combined with open access and open source Wiki-technology approaches*.

The current debate [3,4] thus far suggests that, for recovery of facts from texts, we are dealing with an either/or dilemma. However, nowadays computational analysis of text and the involvement of the expert community in the curation of mined (potential) facts from existing and newly created texts can be combined [15]. The expert community, including the original authors of manuscripts, can be assisted by computational analysis of their newly written text on the fly to suggest the implicated facts. This is not necessarily restricted to new articles, but can be used for each authors' legacy publications as well, with the aim being to go 'from texts to facts'. Similar tools can be used by professional annotators to mine potential facts and curate them based on the original text fragments.

There are a number of key issues to be addressed.

• The continued challenge to recognize individual biomedical concepts correctly in text, especially when the expressions are ambiguous (beyond just genes and proteins).

• Research about whether simple (sentence) co-occurrence of known concepts, linked back to the original text fragment, is sufficient to efficiently recover facts from texts, when combined with expert (community) curation.

• The development of tools and environments to assist massive fact recovery and curation.

One of the systems developed to enable the latter approach, 'WikiProteins', is currently in alpha testing, supported by a consortium in the biological database field [16]. The system builds on existing leading databases such as Unified Medical Language System (UMLS) [17], UniProtKB-SwissProt, IntAct, and Gene Ontology (GO). Such sources have 'authoritative' status in the Wiki, but the registered expert community can add to the information in files copied from these databases in a structured (relational) as well as in free text mode. Systems based on text mining that refer back to sentences in the original, such as iHOP, can be linked into this environment.

We should make a targeted effort to use the institutional repositories for authors to mine the most important factual sentences from (their own and other) papers. Rather than just trying to develop sophisticated tools for user based triplet mining, we should develop simple and rapid online tools to map known expressions in texts on the fly to unique database identifiers (UMLS, UniProt, and Entrez Gene) and present these highlighted on the screen for ease of correction and annotation into triplets conforming to semantic web standards. User addition of new or missed concepts to the underlying terminology system should be made easy. Consequently, BioCreative should focus on tasks leading to more efficient tools for combined computer and community annotation.

### Seán I O'Donoghue and Lars Juhl Jensen: focus on usability for content providers

*Dr Seán I O'Donoghue is a Research Scientist at the European Molecular Biology in Heidelberg, Germany. His background is in structural bioinformatics, and his current research is focused on systems that make bioinformatics data easier to comprehend and use*.

*Dr Lars Juhl Jensen is a Staff Scientist at the European Molecular Biology in Heidelberg, Germany. He has a broad background in computational biology, having worked on diverse topics including genome visualization, pattern recognition in promoter regions, and microarray analysis. His current research is focused on integration of large-scale experimental data, literature mining, and analysis of biological interaction networks*.

BioCreative has helped to improve significantly the accuracy of named entity recognition. This is good news for content creators, such as database curators, who use dedicated text-mining tools. Although there is still scope for improving dedicated tools, we believe that the next major focus for text mining should be to reach a broader audience of content users, namely molecular biologists and biochemists. We believe that the most effective way for text mining to reach content users is to collaborate with content providers, meaning not only publishers of online literature, but also providers of other types of biological data, such as the EBI and National Center for Biotechnology Information (NCBI) data services.

Making text mining more relevant to content providers and end users will require a change in focus - a new paradigm for text mining. In the old paradigm, the main focus has been on increasing accuracy of thesauri and annotated corpora. We believe the paradigm needs to be changed to one that focuses on increasing the usability and practical application of text mining tools. This change in focus also involves shifting from dedicated and monolithic tools toward tools that integrate with other services.

An example of this new paradigm is the use of text mining in STRING (Search Tool for the Retrieval of Interacting Genes/Proteins) [18]. This web resource displays functional interactions derived mostly from databases of pathways and primary experimental evidence. We used text mining to extend STRING by inferring relationships based on the co-occurrence of protein and gene names in literature. Thus, text mining was used as part of a larger integrated system rather than as a dedicated text-mining system. We feel that this is a model for how literature mining can benefit not only researchers dedicated to creating content but also a much broader audience.

### Russ Altman: building a dynamic model of biology

*Dr Russ B Altman is a Professor and Chair of Bioengineering and Professor of Genetics, Medicine, and (by courtesy) Computer Science at Stanford University. His research focuses on biomedical informatics, particularly applied to pharmacogenomics, protein structural genomics, and physics-based simulation of molecular structure*.

As long as biologists write text, bioinformaticians will be faced with the task of extracting information from text for automated analysis. Progress in biological text analysis has accelerated during the past 10 years, and has now become a major recognized subdiscipline of bioinformatics. The challenges for this field are clear: to create tools for extracting relationships from text in order to provide a 'systems-level' view of biological interactions, uncovering unappreciated relationships and new hypotheses; and to create tools to help biological database curators identify critical literature, and associate it with the molecular players (genes, proteins, metabolites, drugs, and so on) that it annotates. Future challenges for biological text analysis will include the automatic extraction of semantic relationships from text in order to build a dynamic model of biology. Indeed, I expect that there will be an exciting competition between human-engineered ontologies and automatically deduced ontologies as the underlying infrastructure for the biological semantic web. Human-engineered ontologies are precise and accurate, but can be brittle and difficult to maintain. Automatically deduced ontologies will be imprecise, but are likely to be robust and amenable to rebuilding. In either case, the availability of a semantic infrastructure will provide the next generation of semantic infrastructure (analogous to the UMLS in the past 10 years) that will allow biological text analysis to make a leap in performance and utility.

### Judith Blake: text mining and its relation to the publishing and curation communities

*Dr Judith Blake is a Staff Scientist at the Jackson Laboratory, Bar Harbor, Maine. She is a principal investigator for the GO Consortium and for the Mouse Genome Informatics (MGI) consortium. Her research interests focus on semantic standards, ontologies and data integration methodologies for genomic, genetic, and phenotypic information*.

Text mining will help to the extent that the biomedical publishing industry adopts standardized terminologies to describe primary objects in the manuscripts accepted. The terminologies especially suitable to text mining include an official gene name or ID, assay type, taxa, tissue, anatomical terms, GO identified terms in a format that can be mined, and synonyms for all of the above. However, text mining will not serve to make biological knowledge more accessible if access to full-text source material continues to be restricted.

An important short-term application for text mining is automatic indexing of publications. The immediate interest would be how this complements or competes with the work of medical subject heading (MeSH) curators. In fact, the work of MeSH curators is opaque, and the text-mining community might initiate a dialog with them to see how their process can be made more transparent and involve the use of community terminologies.

A longer term application would be storage of publications to include external cross-references such as Entrez Gene IDs, UniProt IDs, or GO IDs. Some of these might be provided by authors and some by bioinformatics curators, perhaps in concert with text-mining applications. The salient point of this exercise, however, is that these cross-references would be contained as part of the metadata of the publication.

Some critical steps in this process are as follows.

• Negotiations with publishers to make online content available. This includes packaging supplementary material with the primary PDF and providing general access to full text after some reasonable time, perhaps 6 months. Other advances would include author-provided metadata such as Entrez Gene ID, links to data files in the GEO (Gene Expression Omnibus) repository, and IDs for protein or gene objects that are the main discussion of the paper.

• Negotiations with PubMed Central to publicly provide metadata on the publications available through their resource, including cross-references provided by identified and vetted groups outside the NCBI.

• Negotiations with National Library of Medicine (NLM) to provide cross-references between publications and MeSH and Online Mendelian Inheritance in Man (OMIM) terms.

• Creation of structured digital 'reviews' based on user description of their problem. For example, if asked 'What are the genes studied in regards to cell cycle control?', the return would be a digital automated report that reads like free text and that summarizes the information with links to the publications that supported each statement. This might initially be constructed for a finite set of queries and then extended to include designation of taxon, year, assay, and so on.

BioCreative challenge evaluations can contribute to solving these problems to the extent that they make use of all aspects of existing information to explore the most effective mechanism to text mine existing resources. The challenge also needs to address the incorporation of BioCreative results into the mix with the curation strategies used by major bioinformatics providers such as the model organism databases and UniProt. Up to this point, it seems as if the text-mining challenges have been self-contained and do not actually impact on the way in which curation of biomedical literature proceeds. It would be a major shift in effort if there were greater collaboration between curators and text miners to test and refine text-mining tools that could be more universally deployed for use with biomedical publications.

### Casey Bergman: text mining for extraction of protein and molecular interactions

*Dr Casey M Bergman is Lecturer in Bioinformatics and Functional Genomics in the Faculty of Life Sciences at the University of Manchester. His current research focuses on the genome informatics and comparative analysis of non-protein-coding DNA, with an emphasis on cis-regulatory regions and transposable elements*.

From a general bioinformatics perspective, the performance of text-mining systems to solve 'mature' problems (like the Gene Mention task) is much higher than many other domains of computational biology research. For example, about 85% to 90% of precision/recall obtained by the highest scoring systems in the Gene Mention task are currently unattainable in regulatory bioinformatics [19,20] and approach the highest performing systems in gene prediction [21]. Thus, the time is right to put mature text mining systems into action for biological knowledge discovery and truly integrate 'bibliomics' with other postgenome data sources.

The text-mining community should be looking to build stronger links with the bioinformatics community before looking to the general community of biologists. Researchers in bioinformatics can bridge the middle ground between text miners and biologists, are more likely to be early adopters of text mining technologies, and are able to integrate these systems into other applications or workflows that biologist would be more likely to use. To help bioinformaticians adopt text-mining technologies, there will need to be a greater emphasis on developing text-mining systems that interface with or use open source bio-software systems, such as Bio-PERL.

One short-term application for text mining would be to leverage success from the protein-protein interaction tasks to try to detect other molecular interactions, in particular protein-DNA interactions (transcription factor-target gene interactions). This will require methods to disambiguate gene names used in their protein or DNA contexts, and hints to solve this problem might be captured in the experimental techniques used. A longer term application building on detecting individual protein-protein and protein-DNA interactions would be to develop text-mining systems that automatically assemble interaction or regulatory networks, as the work of Saric, Rodriguez-Penagos, and colleagues has shown is indeed possible [22,23].

For a future BioCreative, I would like to see the protein-protein interaction tasks run again in parallel with a challenge on protein-DNA interactions. It will be critical to run the Interaction Method Subtask or a related challenge again, because only a limited number of teams participated in this task and accurately mining experimental methods will be a key to many text mining applications, including disambiguating protein-protein interactions from transcription factor target gene interactions.

### Christian Blaschke: making text mining results accessible to end users

*Dr Christian Blaschke is Chief Scientific Officer and leader of the text-mining projects in Bioalma, Madrid, Spain. His academic work has been focused on text mining applied to molecular biology and biomedicine, where he has published in the areas of protein-protein interactions, DNA array analysis, and automatic ontology learning. Christian Blaschke was also one of the organizers of the BioCreAtIvE (Critical Assessment of Information Extraction for Biology) challenge carried out in 2004*.

Text mining is not yet able to make biological knowledge more accessible. At present, the influence of BioCreative seems to be restricted very much to the text mining community, with some interest from biological databases; it has not (yet) reached the end users of information.

The text mining community is very data focused. Even if much more information could be tagged reliably, it would still be useless for people who are not text mining researchers. There are two main problems. The first is storing and maintaining the data; large data warehouses are difficult for academics. Second, end users need interfaces and not just the data. Producing the data is not enough; good user interfaces are necessary for biologists to use the results produced by text mining.

Text mining is now at the point where a wide range of entities can be tagged reliably. Thus far, BioCreative has only evaluated gene and protein identification, but a number of groups are also looking at chemicals, diseases, and so on. One possible way to make text mining results accessible could be to negotiate with database providers (for example, UniProt, OMIM, and others) to provide links generated by text mining systems to Medline abstracts. This would enrich these data sources - people could find documents more easily for a given database record - and it would make users more aware of text mining.

Biological text mining still lacks standards at many levels, including at the syntactic level (in what format to express the annotations and how to exchange them) and at the semantic level (what to annotate and how). Currently, BioCreative depends on availability of data and volunteers to set up the tasks by providing both data and criteria to evaluate the results. This makes it difficult for the organizers to select tasks that they think would be most useful for the advancement of the field.

Independent of the specific tasks that are carried out, it is important to make the results more accessible. The idea of a meta-server, discussed at the BioCreative workshop, could be very useful to drive standardization of data interchange formats. It would also make ongoing evaluation (at least theoretically) possible, like EVA for continuous evaluation of protein structures [24], and would be likely to improve coherence on the semantic level too. This could provide an infrastructure in which annotations are made available in such a way that other groups could build user interfaces on top of them. Text-mining researchers are good at analyzing text but are often less good at building interactive systems that users can readily adopt. If a technical solution to making the data available could be found, then other teams might build usable systems on top of that and make the results more visible.

### Hagit Shatkay: user-focused applications for text mining

*Dr Hagit Shatkay is the head of the Computational Biology and Machine Learning Laboratory at the School of Computing, Queen's University, Kingston, Ontario. Her background is in computational biology, statistical machine learning, and databases. Her research spans several areas of biomedical data and text mining, with special focus on the use of text for supporting biological tasks, informative and functional single nucleotide polymorphism selection for disease-association studies, and the integration of text and image data in biomedical applications*.

Given the sheer volume of biomedical information stored in the literature, the wide use that biomedical scientists and database curators make of it, and the laborious process involved in obtaining various types of information from text, there is no doubt that computational text mining methods can - and should - be used to expedite biomedical discovery and curation. The BioCreative results show excellent performance for identifying gene occurrences in text, laying the foundation for other extraction tasks. Other directions in text mining [25], independent of entity extraction, have clearly shown that using text improves performance on a purely biologically motivated task, such as predicting the subcellular location of proteins.

Text mining is not a single method but rather is a large array of tools and approaches, which is a good match for the varied biomedical data needs that also do not form a single well defined problem. To use the mining metaphor, gold-mining requires different tools and is done in different geological regions than coal mining. The key to success - both in mining and in biological applications - is the ability to pair specific problems with the right tools.

For instance, expediting biomedical database curation (for example, in MGI or FlyBase) can be supported by automatically identifying the papers, or even highlighting the paragraphs, that are most relevant to the specific curation task. An information retrieval and text categorization approach can be successfully applied, assuming that the institutes running the database are interested in such a solution, and are willing to provide the needed information to the system developers. A very different application, such as helping a physician to scan the literature for specific gene mutations that have been shown to be associated with an adverse drug reaction, is likely to require the extraction of the gene mentions along with mutation statements and drug reaction facts.

The choice of tools and the acceptable level of performance largely depend on the application, its granularity (are we looking for papers or for statements), and the respective noise tolerance (how many false positives and false negatives can the user tolerate and still view the tool as useful?).

Successful development of biomedical text-mining tools strongly depends on close collaboration between biologists and text miners, in which biologists and medical experts with a variety of research interests identify specific problems that can benefit from using text, and jointly with text-mining researchers address those first. Good candidates are problems for which partial solutions for a subset of the target problem already exist, such as subcellular localization, as in [25]; the latter can serve as ground truth that enables validation without requiring extensive manual evaluation or additional annotation.

To meet the challenges, it is important to keep as much of the data within the articles easily accessible to text miners. For instance, image data within articles is a critical source of information for scientists; as such, it is very likely to be an important component in literature mining [26].

To move biomedical text mining from research to practice, the challenge should focus on posing real biological problems or problems that relate to the construction of current biological resources and databases. The definition of future tasks needs to involve working closely with database curators or with people that are involved in data-intensive applications (from the NLM, MGI, FlyBase, meta-genomics initiatives, and so on) to gain an understanding and a clear statement of their specific text-related data needs. This will make it possible to define tasks and challenges that address a subset of these needs, such as identifying experimental evidence or methods in the literature, finding papers relevant for curation, and addressing specific and well motivated biomedical questions.

### Aaron Cohen and William Hersh: realizing the potential of text mining for biomedical applications

*Dr Aaron M Cohen is an assistant professor at Oregon Health & Science University in the Department of Medical Informatics and Clinical Epidemiology. His research interests focus on the development and application of text-mining techniques and tools for biomedical researchers. He received an MD from the University of Michigan, and holds a Master's Degree in biomedical informatics*.

*Dr William Hersh is Professor and Chair, Department of Medical Informatics and Clinical Epidemiology, Oregon Health & Science University. His research interests include the design and evaluation of information retrieval systems as applied to biomedical tasks. His current research focuses on entity-based question answering in the genomics domain and medical image retrieval*.

The recent BioCreative challenge results demonstrate that we have reached the point at which text-mining tools can help to make the biomedical literature, and therefore the knowledge base of biology and medicine, more useful and accessible. The performance of basic algorithmic tasks such as named entity extraction, entity normalization, and protein-protein interaction are mature enough for these technologies to be beneficially incorporated into user systems. However, it is not clear that a level of accuracy useful for purely automated systems has been achieved and, for the time being, these algorithms must realistically serve as an aid to human curation and information retrieval, and not a replacement for them.

Human-curated databases such as NCBI's Entrez, Jackson Laboratory's MGI, and UniProt/SwissProt are used frequently by working scientists to help them in their research. The collection of this data is expensive and time consuming. Text mining based tools can decrease the per-curated-fact time and cost by allowing curators and other research scientists to focus on the most likely useful and novel source material, and also by improving the interfaces of the tools with entity highlighting and suggested annotations. Computer-aided curation has already proven useful in maintaining the Medline database [27]; other work has shown the potential of this approach in the genomics domain [28,29].

Several things need to happen to make this a reality. First, algorithms must be incorporated into retrieval, curation, and annotation systems on a wide scale. This will require the participation of scientist and curator users and will help focus text-mining efforts on integrating and refining the technology in a way that provides maximum benefit to the support of scientific discovery.

Second, basic text-mining resources, such as domain-specific thesauri and lexicons, need to be developed and shared across research groups and curation tasks. Although there is currently widespread sharing of gene names, synonyms, and functions (for example, Entrez and GO), this is less true for other concepts such as terms describing protein-proteins interactions and species-specific phenotypes. The creation and expansion of these resources will extend both the depth and breadth of the information that can be curated, searched, and data mined.

Third, full text needs to be made more widely available in a machine-readable manner. Although titles and abstracts are widely available and have high information density, much of the knowledge of science remains buried in the full text of journal articles [30], waiting for computer-assisted curation to uncover and catalog it. While most universities have access to full text in the form of HTML and PDF files, these are less than optimal for text processing. The Open Text Mining Interface (OTMI) [31] goes some way in providing a consistent format for text mining. However, typically OTMI is limited to processing text in sentence units. A means to relate sentences to each other and to section header information is needed as well.

Finally, there needs to be more attention paid to user-oriented (extrinsic) evaluation that assesses the value of these tools for realistic tasks and settings. This starts with building better test collections but ultimately must culminate in studies that demonstrate explicit value for these tools. Working biologists and curators should have a strong influence on both the tasks to be algorithmically enhanced and the tunings of the algorithms for best performance in the real world. Challenge evaluations, such as BioCreative, and the TREC Genomics Track [32-35] can help to make this happen. By involving working biologists and curators, evaluations can be designed with specific tasks in mind, and performed within the context of actual curation tasks and real-world information needs. Frameworks can be used that allow different algorithms to be 'plugged in' and used for actual research and curation. The result of this will be real working systems, optimized by the best available algorithms and tunings. These will enhance the ability of curators to annotate and scientists to retrieve a wider range and larger volume of useful biomedical knowledge, increasing access to everyone.

### Dietrich Rebholz-Schuhmann: the limitations of BioCreative - representing biological information

*Dr Dietrich Rebholz-Schuhmann is Group Leader of a research group in biomedical information extraction at the European Bioinformatics Institute (EMBL-EBI), Hinxton, Cambridge, UK. His background is in medicine and computer science. His current research focuses on text mining, integration of literature into the infrastructure of biomedical data resources, and the use of ontological resources for knowledge discovery*.

Indirectly, BioCreative improves access to biological knowledge extracted from the scientific literature, because it bundles biomedical text mining efforts toward the shared goal of trustworthy extraction methods. Directly, BioCreative itself in its current setup does not provide any text mining solution, but this could change in the future (funding permitting). However, BioCreative does two things very efficiently: define new use cases for information extraction and information retrieval, and then measure the performance of the proposed solutions.

There will never be a single 'killer application' for text mining in the biomedical domain, because a high diversity of facts forms the domain knowledge (for example, descriptions of biomedical phenotypes and of experimental conditions to perturb the phenotypic states) and users have different needs. Over time, the bioinformatics text mining community will need to get better access to more content, to work together with the publishers, to develop more efficient tools, and to ask biological users to contribute to the electronic representation of biomedical knowledge (for example, structured digital abstracts).

For me, the key question is, can the biomedical text mining community capture biomedical domain knowledge in terms of its representation of information, for example linkage of named entities to bioinformatics data resources [36]. For extraction of facts from the text, we have to find the means for the representation of information such that a biologist can deal with uncertainty, similar to *p*-values in BLAST. I am not sure whether biologists are ready for this. They want to read the scientific text to make up their minds what the text conveys and to squeeze out all contained truth. The more we move toward better semantic representation of information, the higher the chances that we will be successful on this issue.

The next BioCreative should move toward coverage of more semantic types in its assessments, for example, diseases, chemical entities, and experimental conditions. For genes and proteins, the task could be not only to identify and normalize these terms, but could be extended to handle complete syntactic structures that modify the semantic interpretation, as in concepts like ' [protein] activity', 'the expression of [protein]', 'the mutant of [protein]', and ' [protein] promoter region', where [protein] stands for a named entity denoting a protein or gene. This would require ontological knowledge to interpret term representations and solutions for ontological term mapping, similar to the identification of GO terms in text.

### Udo Hahn: directions for future BioCreatives

*Professor Udo Hahn is the head of the Jena University Language & Information Engineering (JULIE) Laboratory in Jena, Germany. His research focuses on biomedical information extraction, text summarization, and text mining incorporating both advanced human language technology infrastructure and ontologies for the life sciences*.

As already witnessed by other types of human language technology competitions (for example, for information extraction [MUC [37], ACE [38]], document retrieval [TREC [32]], or text summarization [SUMMAC [39]]), we also observe for BioCreative a steady increase in the accuracy and quality of results for tasks that are run continuously over several single competitions. There seems to be a strong tendency for the involvement of internationally leading research groups, when working on the same problem types over time, to generate substantial, empirically measurable progress in terms of the quality of system outputs. Hence, BioCreative-style competitions (currently) are productive for methodological consolidation and improvement in biological information extraction. Consequently, they have already rendered biological knowledge explicit and thus accessible (in the form of named entities in particular) that might otherwise have remained locked away in the mass of publications that rely on purely intellectual efforts of biological researchers and database curators only.

BioCreative, up until now, has focused on gene name recognition and normalization with respect to named entity tasks, and on protein-protein interactions with respect to relation extraction. Both topics are crucial for molecular biology but certainly leave room for alternative issues that are similarly relevant or even more exciting. Hence, future BioCreatives might consider additional biologically relevant entities such as chemicals, diseases, species, and (OBO-Foundry-style [40]) relations that they are involved in, such as *derives from*, *located in*, *has agent*, or *has participant*. Of particular long-range interest from the perspective of ontologies are models of biological events and their representation as partially ordered subevents, for example, gene regulation and expression, or variation and mutation events. As an additional axis of description, species-dependent information should be determined, for example, for the various model organisms and findings specific to them.

Thus far, BioCreative has been more a (quite restricted) information extraction task rather than a true text mining task, the latter being characterized by helping to shape or focus on interesting, relevant, new, or controversial biological knowledge. This is most clearly indicated by the protein-protein interaction (PPI) subtask from the second competition. A particular restriction was made to limit the system output to a (binary) pair of proteins involved in the PPI, a fairly common constraint in many relation extraction experiments. Often, many of these protein pairs are either already known to skilled biologists (and hence irrelevant) or are rather controversial because of inconclusive experimental data. Future BioCreatives should therefore broaden their perspective to uncover information from publications that might be referred to as 'additional constraints' or 'contextual factors' on, for example, PPI findings such as various experimental setups (for example, detection procedure, and media and materials being used), under varying experimental conditions (degrees of pressure, temperature, or humidity), the time lines of experimental effects, the statistical methods being applied to the available experimental dataset, and so on. With such additional information being made explicit, the often cautious claims made by the authors in certain publications could be grounded in considerations that are entirely discarded by merely binary PPI data. Furthermore, because such data might provide positive as well as negative evidence for some PPIs (and other types of biological events), this might truly serve as a resource repository from which new and interesting biological knowledge could emerge, either already automatically identified or merely as a focusing mechanism heuristically guiding biologists to controversial research issues. One could even imagine that such value-adding services might result from applying embedded reasoning mechanisms (inference rules) such that implicit knowledge (assumptions, gaps, contradictions, and so on) is made explicit and, hence, more readily exposed to further assessment of domain experts. This then might lead BioCreative along the road to real text mining.

Major parts of this information will only be accessible from full-text sources. Hence, BioCreative should further emphasize the role of and access to the original full-text rather than its content-wise limited derivatives, e.g., (Medline) abstracts. Note that this potential extension does not come for free. Full-text documents (other than abstracts) are characterized by a large variety of complex text cohesion (various sorts of pronominal, nominal, subgrouping and bridging anaphora, as well as local, spatial, or temporal forms of reference) and text coherence phenomena (rhetorical and argumentation structures, causality, and evidence relations), which establish connectivity among sentences, and the propositions they encode, at the micro and macro levels of text composition. Turning our attention to a broader range of textual material, we might also consider not just published material from prestigious, peer-reviewed journals but also material scattered over the web, for example, Wikipedia-type sources, conference proceedings, text books, blogs, mailing lists, and so on.

BioCreative, intended to serve the pressing needs of the biology community, should certainly not lose sight of the biology researchers' routine work environment. Hence, the functional annotation task (only run in the first BioCreative competition, although with limited success) should not get excluded from future BioCreatives but rather should be revitalized and redefined. In particular, the comparison of automatically generated annotation data with intellectually generated ones (originating from human database curators) and the spotting of annotation gaps (given automatically generated data) could render entirely new services to the biology community. An additional task of greater impact for biologists could be the automatic generation of pathways from literature input, which has been a manual activity up until now.

Besides well covered information extraction scenarios, future BioCreatives should also turn to additional human language technology services and functionalities not considered thus far, such as the automatic summarization of biological knowledge from large compilations of full-text documents or question-answering-type applications. Finally, relations to neighboring disciplines might also be taken into account to a greater extent. Of particular relevance are the genotype-phenotype linking that relates molecular biology to medical research in terms of diagnoses and therapies for several diseases and pathological states in organisms, and the genotype-drug design linking, which relates molecular biology to pharmaceutical and chemical research and development.

If future BioCreatives are to tackle at least some of these additional tasks and functionalities, then heavy investment in the infrastructure of these competitions must be made. This holds, in particular, for the creation and maintenance of large-scale and (unlike today) diversely annotated corpora (both in specification depth and domain coverage), for which combined international efforts may be required. As a side effect, such large-scale annotation efforts could only be reasonably run if a wide range of sophisticated, stable, and sharable annotation tools (including annotation language definitions and annotation guidelines) were available. It is currently entirely speculative (and perhaps worth a comparative study within the context of some future BioCreative tasks) whether such tools kits should be distributed directly (and hence be specifically adapted) to the life science community. This would empower the emergence of community-based annotations and edits, such as social annotations, perhaps even based on biological folksonomies or, more precise, 'expertonomies', thus possibly breaking up the often deplored annotation bottleneck. Another major nontechnical infrastructure issue will be the supply of more in-depth links between these annotations, interlinked biological ontologies (which capture general biological knowledge), biological lexicons and terminologies (which capture, among other information, the different synonyms of biological names), and biological databases (which keep the concrete assertional, empirically determined biological knowledge about specific biological entities and their interrelations). Under these unifying conditions, the mediating and central role of biological ontologies for manual as well as automatic knowledge management in the life sciences would become even more apparent, far beyond the current main use for functional annotation.

## Conclusion

*Dr Lynette Hirschman is Director, Biomedical Informatics at the MITRE Corporation in Bedford, Massachusetts, USA. Her background is in natural language processing and, more recently, bioinformatics. She is a founder and co-organizer of the BioCreative text mining challenges. Her current research focuses on text mining for the biomedical domain, bioinformatics, and capture of metadata for genomics and metagenomics*.

*Martin Krallinger is currently working at the Spanish National Cancer Center in the Structural Biology and Biocomputing Department. Previous research stays included the National Biotechnology Center (CNB, Madrid, Spain) and the Center of Applied Molecular Engineering (CAME, University of Salzburg, Austria). His main research interests are related to text mining, information extraction and information retrieval applied to the biomedical and molecular biology literature. He is a co-organizer of the most recent BioCreative text mining challenge evaluation*.

*Dr Alfonso Valencia is Director of the Structural and Computational Biology Programme at the Spanish National Cancer Research Centre (CNIO), Madrid and Director of the Spanish National Bioinformatics Institute (INB). He is Executive Editor of Bioinformatics (Oxford University Press), and a founder and member of the board of the International Society for Computational Biology. He is also a founder and co-organizer of the BioCreative text mining community challenge*.

The contributions from the previous sections touch on many broad issues of critical importance to our understanding of how text mining can provide better access to the biological literature. In conclusion, we wish to highlight some of these issues, because they will occupy 'center stage' in our community discussions as we move forward.

### Ontologies and text mining

Ontologies and controlled vocabularies occupy a central role in text mining; they provide the set of categories used to label or distinguish different types of entities, data, or relations. However, we do not understand well the relationship between ontologies and natural language processing. Some basic aspects, such as human annotation consistency and the importance of experimental evidence types, have not yet been studied in detail. Also, there are many open questions.

• How can ontologies, which have been designed primarily for enabling consistent models of biological knowledge, be used for describing/representing biological information contained in the literature?

• What is the connection between the ontologies with their definitions and synonyms, and the kind of lexicon that is useful for natural language processing?

• How can the structure of ontologies be used to enhance text mining?

• How can text mining or natural language processing be used to automate the construction of ontologies? In particular, how can term extraction tools for the biomedical domain be used to assist in the development of controlled vocabularies to be integrated into biological ontologies?

### Curation and biological databases

It would be useful if the curation effort itself were more formalized and if it were monitored in terms of time spent per curation step. An analysis of the main sources of annotation errors would also be helpful, as would more extensive inter-annotator agreement studies. This would provide the biological database community with an opportunity to point out where text mining could improve curation in terms of efficiency (time) and consistency (accuracy). Current annotations also lack pointers to the evidence passages supporting the annotation, which makes it difficult for researchers to assess the underlying evidence and to interpret the annotations. Linking evidence passages to annotations would provide valuable training data for the development of text mining tools as well as making interpretation and update of annotations easier.

Databases can be viewed as containing a summary of the available information. To define candidate tasks for future text mining efforts, it would be useful to know, for example: what are the main kinds of information that are biologically relevant but missing in current curated resources? What are the main categories of information that cannot be collected with current text mining methods? What are the limitations on granularity of information that can be collected?

### Document processing and structure

An understudied area is document preprocessing, including document structure, text formatting, and the general preprocessing necessary before text mining techniques can be applied. There are some initiatives on processing noisy texts, such as those that result from conversion from PDF to ASCII, in other domains. Full-text processing starts with conversion into plain text, which has a significant effect on all the 'down stream' text mining, starting with text tokenization and entity recognition. In addition, full text articles have a complex (and variable) structure, including headings, figure legends, tables, and so on. It is important that text mining systems begin to take advantage of this structure, to locate important information or to ignore distracting information (for example, mentions of gene names or diseases in titles of articles in the bibliography). We are just starting to see research that can exploit this rich content in innovative ways. In the longer term, it may be appropriate to couple text mining research with image classification and image understanding to full exploit these features.

### Evaluation

There are many open questions on evaluation. What is 'better' information access? How do we measure this? How do we evaluate interactive interfaces, where the user and system work together to accomplish a task? How can we define evaluations that scale to realistic tasks? How representative are test collections in terms of the existing or widely used text repositories considered by curators? What are the best ways to present and visualize the results of these comparisons? Can we combine results from multiple systems to provide improved performance? How informative are the scores, and can these scores help biologists to combine information from text mining with other information sources? Only collaborative efforts between both the general users of text mining tools, as well as specialized users such as database curators, can provide design of realistic tasks, resulting in real applications to improve information access. The lessons learned from CAFASP (Critical Assessment of Fully Automated Structure Prediction, for structural bioinformatics [41]) could provide interesting insights here.

### The triangle: publications, databases and users/developers

We see that publications are increasingly found online in computable digital format. Meanwhile, databases are struggling to keep up in their efforts to extract data and biological information, and users/developers constantly need to combine information from databases and publications to progress in the interpretation of their own results.

In this triangle of interlinked activity, the first issues are related to the interoperability of the text resources. There is ongoing discussion at many levels about how to address these needs. Various initiatives are emerging for the creation of text repositories, including the many legal and technical issues. Also, there is active discussion on how these repositories will be organized to facilitate research and exploration by text mining technology.

The second key step is the creation of digitally annotated abstracts, a pioneering effort to introduce structure into the text that can be fed directly to the databases. This is a complex scenario that will require the collaboration of editorial houses, editors, authors, and databases, with a careful definition of mutual benefits. Questions related to the accessibility and coherence of the annotations, validation of the annotations, and the economics of the process will have to be carefully assessed along the way.

Finally, the challenge for the text-mining community is to understand the role of text mining in this environment of digitally annotated abstracts, and to offer realistic approaches and integrated tool suites.

The ultimate goal motivating the BioCreative undertaking is to put text-mining tools to work. In addressing the challenges outlined here, we believe that the applicability of text-mining tools will broaden, their performance will improve, they will become more easily embedded in the workflow, and - the ultimate success - text mining as a 'special capability' will become invisible as it becomes a routine part of the tool box of the bioinformatician and biologist.

## Abbreviations

EBI, European Bioinformatics Institute; GO, Gene Ontology; iHOP, Information Hyperlinked Over Proteins; MeSH, medical subject heading; MGI, Mouse Genome Informatics; NCBI, National Center for Biotechnology Information; NLM, National Library of Medicine; OMIM, Online Mendelian Inheritance in Man; PPI, protein-protein interaction; SFI, Science Foundation Ireland; UMLS, Unified Medical Language System.

## Competing interests

The following authors declare that they have no competing interests: Russ Altman, Casey M Bergman, Judith Blake, Aaron Cohen, Les Grivell, Udo Hahn, William Hersh, Lynette Hirschman, Martin Krallinger, Lars Juhl Jensen, Seán I O'Donoghue, Dietrich Rebholz-Schuhmann, Hagit Shatkay, and Alfonso Valencia.

Christian Blaschke's work was funded by Bioalma. Frank Gannon, Director General of SFI, declares that he has no competing interests; SFI as a funding agency supports work in a variety of groups that are active in the area of software development. Dr Gannon's research group has in the past been supported by Wyeth Pharmaceuticals. Dr Gannon is the founder of three companies in areas unrelated to this topic. Manuel Peitsch is listed as an inventor on a Novartis patent for the Ultralink (USPTO Patent Application #: 20060150087; Patent Title: Ultralink text analysis tool.) text analysis tool; his work has been supported by Novartis AG. Barend Mons' work was supported (in part) by the company Knewco.

## Authors' contributions

Authors are listed in alphabetical order. MK and AV were responsible for inviting contributors; LH assembled and edited the collection of opinions. LH, MK, and AV were responsible for the Conclusion section. The authors contributing opinions are RA, CMB, JB, CB, AC, FG, LS, UH, WH, LJJ, BM, SIO, MCP, DRS, and HS. The participation of LH (MITRE) was supported under National Science Foundation Grant II-0640153.
